# Investigating the Applicability of the SAFER‐YCL Care Bundle for Transitions From CAMHS Crisis and Liaison Services: The Barriers and Enablers

**DOI:** 10.1111/hex.70579

**Published:** 2026-02-02

**Authors:** James Roe, Neve Jones, Leonie Lewis, Natasha Tyler, Maria Panagioti, Sewanu Awhangansi, Nisha Balan, Nicola Wright, Richard Morriss, Kapil Sayal, Pallab Majumder, Josephine Holland

**Affiliations:** ^1^ National Institute for Health and Care Research, Applied Research Collaboration (ARC) East Midlands University of Nottingham Nottingham UK; ^2^ Mental Health and Clinical Neurosciences, Institute of Mental Health University of Nottingham Nottingham UK; ^3^ Nottinghamshire Healthcare NHS Foundation Trust Nottingham UK; ^4^ Division of Population Health, Health Services Research and Primary Care, National Institute for Health and Care Research Greater Manchester Patient Safety Research Collaborations, NIHR School for Primary Care Research University of Manchester Manchester UK; ^5^ Leicestershire Partnership NHS Trust Leicester UK; ^6^ School of Health Sciences University of Nottingham Nottingham UK

**Keywords:** CAMHS, clinical practice, Crisis and Liaison services, discharge, mental health, transition, youth mental health

## Abstract

**Background:**

Crisis and Liaison teams in Child and Adolescent Mental Health Services (CAMHS) offer intensive, short‐term support to young people experiencing mental health crisis in the community (Crisis) or admitted to acute hospitals (Liaison). There is no evidence‐based model for how these teams operate. The SAFER care bundle, designed to improve discharges from acute hospitals, has been adapted for use in mental health inpatient discharges for adults (SAFER‐MH) and young people (SAFER‐YMH). This study took a care bundle designed to improve discharges from CAMHS inpatient care (SAFER‐YMH) and used stakeholder feedback to adapt it for use in CAMHS Crisis and Liaison teams.

**Design:**

Focus groups were carried out with healthcare professionals (HCPs), young people and parents/carers to present the SAFER care bundle and discuss necessary adaptations for use in CAMHS Crisis and Liaison teams. Analysis of transcripts followed a Normalisation Process Theory (NPT) framework to identify barriers and facilitators to implementation and necessary adaptations.

**Results:**

Participants expressed that integrating the SAFER‐YCL care bundle into the electronic patient record, automatically pulling information from other forms, and providing a template for discharge letters and safety plans could serve as an aide‐memoire and potentially replace current discharge documents. It would need to avoid increasing documentation burden for staff and have flexibility to be administered by different staff members and at an appropriate time.

**Conclusions:**

The SAFER‐YCL care bundle has been successfully developed for implementation in CAMHS Crisis and Liaison services, demonstrating potential to enhance transition experiences. Feasibility testing will be crucial to validate its effectiveness and facilitate successful integration into clinical practice.

**Patient or Public Contribution:**

This study was initially presented at the Nottinghamshire Healthcare NHS Foundation Trust's Involvement group of young people to gather their thoughts on it. They were supportive of the study design and gave constructive feedback on the study. A PPI representative with lived experience was part of the study team who was involved in developing and reviewing all study materials, was part of monthly reviews of the study's progress and supported data collection, analysis and write‐up of the study results.

## 1 Introduction

It is estimated that one in seven children and young people (CYP) globally experience mental health difficulties [1, 2], a figure which has been rising over time [3]. Consequently, Child and Adolescent Mental Health Services (CAMHS) are experiencing increased referral rates [4] and increasingly severe and acute presentations [5, 6, 7]. The rise of mental health crises [8] is putting strain on intensive support services like inpatient mental health care, paediatric wards, emergency departments and police departments [5, 6, 7, 9]. CAMHS Crisis services are community teams providing short‐term, intensive support for young people (YP) experiencing mental health crisis, aiming to reduce hospital admissions and forming a key part of the NHS long‐term plan for CAMHS in England [10, 11]. CAMHS Liaison teams offer mental health support to YP within acute hospitals. In some regions, these teams collaborate due to their similar, short‐term, intensive service model. There is no evidence‐based model for their operation [10], so provision varies between countries, regions and organisations. These teams often work 24/7, staffed by experienced mental healthcare professionals (HCPs). Referrals may come from YP/carers, primary and secondary care or other services (e.g., police). YP are typically discharged from these teams to less intensive community mental health services or primary care. These teams can support far more YP than inpatient care and referrals to CAMHS Crisis are increasing [12]. To meet the growing demand, efficient patient flow is essential, ensuring YP are safely transitioned to appropriate services in a seamless and timely manner. Transitions are often a stressful process in patients' care journeys, with potential for gaps in effective information sharing [13], tension between services and agencies, long wait periods and the risk of being lost from care services. Patients and their carers often navigate complex, fragmented systems without adequate support or information [13]. Smoother transitions can improve care experiences for YP, families and clinicians, and potentially patient outcomes. Research shows that simpler interventions and co‐production with users are often more effective when designing interventions to improve healthcare transitions [13, 14]. The NHS Improvement SAFER patient flow care bundle is a practical framework designed to improve discharge and transition from inpatient settings to community services [15, 16]. It is a set of operational practices to facilitate patient flow and avoid unnecessary delays with discharge. This has been adapted for use in adult inpatient mental health settings (SAFER‐MH) and adolescent mental health inpatient settings (SAFER‐YMH) [17]. The SAFER‐YMH bundle consists of a patient co‐created (written) discharge plan, to encourage better information sharing and shared decision making, a social information capture form for gathering and acting upon critical social information about the patient, and standardised checklists to streamline the discharge process. The feasibility of these care bundles has been recently tested in inpatient services [17]. However, the applicability of the bundle for transitions between more intensive and general community services has not yet been explored. Crisis and Liaison teams differ from most other community mental health services as they are designed to offer shorter term, intensive support to manage mental health crises, address high‐risk situations and reduce the need for inpatient admission. Discharge from these services therefore has many parallels to discharge from inpatient services, requiring the same level of careful planning to effectively manage high‐risk, complex patients. The purpose of this study is to investigate the applicability and necessary adaptations of the SAFER‐YMH care bundle for further testing in a feasibility and acceptability study within CAMHS Crisis and Liaison teams facilitating transitions from these services.

## 2 Methods

### 2.1 Aims

1. To identify the barriers and enablers, as well as necessary adaptations for implementing the SAFER‐YMH care bundle in CAMHS Crisis and Liaison teams.
2. To produce a co‐adapted version of SAFER‐YMH care bundle that is ready for implementation in CAMHS Crisis and Liaison teams.

### 2.2 Study Design

Two phases of focus groups were carried out with HCPs, YP and parents/carers (PCs). Phase 1 focus groups involved presenting the SAFER‐YMH care bundle and discussing the necessary adaptations for its use in Crisis and Liaison services. Phase 2 focus groups involved presenting an adapted version of the SAFER‐YMH care bundle based on findings from Phase 1, and discussing any further adaptations needed for implementation.

### 2.3 Participants and Setting

Participants were either healthcare and support professionals (clinicians, managers and IT professionals), YP or parents of YP with experience of engaging with CAMHS Crisis and/or Liaison services. Focus groups were conducted at two mental health trusts (provider organisations) in England. Where possible, focus groups with healthcare and support professionals took place with individual CAMHS teams to obtain a wide range of professional roles of those who work in, and with, Crisis and/or Liaison services.

### 2.4 Procedures

Ten Focus groups, lasting between 30 and 60 min, were facilitated by the lead researcher with extensive qualitative research experience (J.R. [male research fellow, PhD]) and one of the study co‐investigators (N.J. [female research assistant, BSc], J.H. [female consultant psychiatrist, MA(Oxon)], L.L. [female academic foundation doctor, MBChB] or P.M. [male consultant psychiatrist, PhD]), and were carried out either face‐to‐face (*N* = 4) or online (*N* = 6) between February 2024 and August 2024. Participants gave informed written or electronic consent. Focus group discussions were audio recorded, transcribed and pseudonymised after completion.

Topic guides were co‐produced with Patient and Public Involvement (PPI) input and underpinned by Normalisation Process Theory (NPT) allowing for a detailed exploration of how interventions, practices and procedures come to be part of routine practice within an organisation [18]. Four constructs make up NPT: coherence (understanding and sense making), cognitive participation (who uses the intervention and needs to be involved), collective action (what needs to be done, where and when) and reflexive monitoring (evaluation and monitoring of the benefits and harms).

#### 2.4.1 Phase 1 Focus Groups

During Phase 1 focus groups (3 groups, *n* = 22), the researchers presented the current SAFER‐YMH care bundle (see Supporting File S1) to a range of participants to obtain feedback on where this care bundle would sit within CAMHS Crisis and Liaison teams' current practice and what adaptations would be required to utilise the care bundle with optimum efficacy within these services. Phase 1 consisted of two face‐to‐face focus groups with HCPs (*n* = 11 and *n* = 9), and one online focus group was carried out with YP (*n* = 2) to explore their perspectives on the care bundle.

#### 2.4.2 Phase 2 Focus Groups

During Phase 2 focus groups (7 groups, *n* = 33), the researchers presented the adapted SAFER‐YCL care bundle to a range of participants to obtain feedback on how the adapted care bundle would integrate with current practices and any further adaptations required to successfully implement the care bundle within services. Phase 2 consisted of two face‐to‐face focus groups with HCPs (*n* = 10 and *n* = 7), four online focus groups with HCPs (*n* = 3 per group), and one focus group was carried out with YP (*n* = 2) and PCs (*n* = 2) to explore their perspectives on the adapted bundle.

### 2.5 Analysis

Focus group transcripts were coded in NVivo12 via a deductive framework analysis approach. Initial codes were grouped into broader themes within the general categories of barriers, enablers and required adaptations in line with the central research questions. Inductive analysis for data that did not fit within the deductive framework was also carried out. Themes derived from the data were also mapped onto key NPT constructs. Themes were developed by J.R., N.J. and L.L., then discussed and agreed within the wider research team. One researcher (J.R.) coded all the data. Two researchers (N.J. and L.L.) independently coded four transcripts (~44%) for cross‐checking.

## 3 Results

In total, 49 people took part in the study. Characteristics of study participants can be found in Table 1. Four themes relating to barriers to the implementation of the SAFER bundle were identified (Prevention of Duplication, Time and Place, Continuity Issues and Raised Expectations). Three themes were identified relating to enabling factors (Aide‐mémoire, Integration of other forms and Integration into the electronic patient record [EPR]). Four themes were identified under required adaptations (Crisis and Liaison focus, timescales, optional elements and simplicity). Themes and related NPT constructs are presented in Table 2.

**Table 1:** Participant characteristics.

Characteristic	*N* (%)
*Gender*	
Male	12 (24.49%)
Female	36 (73.47%)
Prefer not to say	1 (2.04%)
*Ethnicity*	
Non‐White	12 (24.49%)
White: English, Welsh, Scottish, Northern Irish or British	33 (67.35%)
Prefer not to say	4 (8.16%)
*Age*	
Under 25	6 (12.24%)
25–34	14 (28.57%)
35–44	13 (26.53%)
45–54	6 (12.24%)
55–64	8 (16.33%)
Prefer not to say	2 (4.08%)
*Participant type*	
Healthcare professional	43 (87.76%)
Young person	4 (8.16%)
Parent/carer	2 (4.08%)

**Table 2:** Themes and related NPT constructs.

	Theme	NPT construct
Barriers	Prevention of Duplication: Upgrade or replacement	Coherence/cognitive participation
	Time and Place	Cognitive participation/collective action
	Continuity Issues	Cognitive participation/collective action
	Raised Expectations	Cognitive participation/collective action
Enablers	Aide‐mémoire	Coherence
	Integration of other forms	Collective action
	Integration into electronic patient record (EPR)	Collective action
Adaptations required	Crisis and Liaison focus	Coherence/cognitive participation
	Timescales	Cognitive participation/collective action
	Optional elements	Cognitive participation/collective action
	Simplicity	Cognitive participation/collective action

### 3.1 Barriers

To facilitate the implementation of the SAFER‐YCL bundle into practice, participants identified several potential barriers to overcome.

#### 3.1.1 Prevention of Duplication: Upgrade or Replacement

Participants repeatedly identified several systemic issues, initially coded as repetition and additional work, which were later grouped under a theme of prevention of duplication as a barrier to implementation. Despite most HCPs understanding the principles of the SAFER bundle and largely valuing its objective, the significant overlap or similarities between the data collected as part of the bundle and existing documents on the system were a cause for concern; HCPs did not want the additional burden of documenting the same information twice.

Adding in something that's just duplicating what's already there isn't going to be of any benefit to the patient journey and it's just going to irritate the clinicians.

(Mental Health Nurse—Crisis Team)

Some viewed the proposed forms as an improved version of their own data collection processes and could envisage how the bundle could supersede and combine current assessments to achieve its aim:

I think once we agree this, this is going to re‐inform our assessment forms that we have, just to make it easier for practitioners.

(Crisis Team Leader)

#### 3.1.2 Time and Place

The theme of time and place emerged as multiple HCPs repeatedly questioned whether the Crisis and Liaison teams were the most appropriate team given the context within which they operate to collect information for the bundle. They felt the relationship to be established with the YP/PC, and the time needed for that, to complete the bundle was difficult to attain. In addition, some HCPs wondered whether YP in a mental health crisis would be too distressed to meaningfully participate in the co‐production elements of the tool.

As Crisis and Liaison teams provide intensive short‐term engagement, in contrast to inpatient or community‐based teams, concerns were raised whether Crisis and Liaison teams were best placed in this setting:

It's a problem because you don't have that relationship sometimes to get all the information.

(Mental Health Nurse—Crisis Team)

The perceived acuity of Crisis and Liaison presentations was considered higher than what community or inpatient teams typically manage, leading to challenges in fully understanding and connecting with YP and their primary caregivers.

You see the patient twice and then you might never see them again. See them twice for like one hour each time, and that's it, if that. Sometimes you see them once and then you speak with them on the phone, and that's it, so you literally just saw them once.

(Mental Health Nurse—Liaison Team)

HCPs questioned whether, shortly after a crisis situation and when YP is distressed, they would be calm and reflective enough to offer this type of information in the timescales required. This led to an adaptation emphasising the completion of the bundle at the earliest appropriate time rather than a specific time period such as 48 h:

I'm just wondering whether in a crisis situation when we first see them, when they're so distressed, whether they'll be able to actually have that calm and reflective, safe environment.

(Mental Health Support Worker—Liaison Team)

Concerns were also raised that YP and PCs could become distressed when asked to help complete such a comprehensive document at that time. As such, preventing future admissions or crisis may seem premature if the YP is still dealing with a current episode so the completion of the bundle may be more appropriate once the current crisis has subsided:

What will help me prevent another admission? I think if you give that in an acute setting, I don't know how parents will take it, or the young person take it, they'll just get upset.

(Mental Health Nurse—Liaison Team)

This was echoed by some YP who also questioned whether they would be able to complete reflexive aspects of the bundle due to their situation at the time:

I still didn't know that stuff, how would I know if I'm better?….I don't think there was anything that could have been done to make you be able to do that, because if you don't know you don't know.

(Young Person)

#### 3.1.3 Staff Continuity and Conflict

Due to the 24/7 nature of these teams, staff work shifts and have a shared caseload. Some staff highlighted that this style of working may mean it is difficult to build enough rapport to complete the forms:

I'm just aware I guess within the crisis team that it will be different clinicians that will be meeting with the young person, so in terms of continuity of completion of this and those conversations around it, that might just be a complicating factor with it.

(Psychiatrist—Liaison Team)

HCPs also queried whether the bundle could have the potential to cause conflict between services. For example, as the bundle asks for details of the YP's next appointment with social care, education and so forth, this could lead to unrealistic expectations:

I think that would cause conflict as well, because you'd get school going, you're trying to criticise us because we haven't got another appointment. That might cause a little bit of angst.

(Psychiatrist—Liaison Team)

#### 3.1.4 Raised Expectations

Similarly, overt sections on medication and diagnosis were viewed negatively by clinicians as this was perceived to appear to YP/PCs as an option they could have, thus raising expectations that they will receive either or both:

Personally, I think including a big section on medication might not be helpful, sets up a bit of an expectation. Maybe a prompt and then have this as an add‐on.

(Mental Health Support Worker—Liaison Team)

### 3.2 Enablers

#### 3.2.1 Aide‐Mémoire

Most staff and YP/PCs were clear in their understanding of the aims and purpose of the SAFER‐YCL bundle to support transition out of CAMHS Crisis and Liaison. Many HCPs could see a value and benefits, such as giving an overview of relevant information about a young person, identifying triggers, acting as information for signposting or handover:

It just gives a little bit of a snapshot about all sorts that you might miss if you're just doing a quick handover. You might forget to say that parents are struggling financially, and that's having an impact, and stuff like that. You might forget those bits. I think it's usually things that we would have usually covered, but then the care coordinator, if they haven't spotted it in the progress notes would actually do it again. This actually will be really helpful for those things to be highlighted.

(Mental Health Nurse—Crisis Team)

YP and PCs also identified value in the bundle and its capacity to give an overview of key information:

I think this kind of thing, having a form there that's got phone numbers on it, it's got contacts, it's got appointment dates, brilliant. Just to talk through, as a parent just to talk through what your, which we did I think, what my anxieties were, what I was worried about. To have it like that just seems like a really good idea, it's common sense to me.

(Young Person)

#### 3.2.2 Integration of Other Forms

As mentioned above, HCPs highlighted issues with the successful completion of the bundle due to the nature and context within which Crisis and Liaison services operate. This was largely attributed to issues such as the timing of engagement and duplication concerns.

To counter these barriers, thus making the use of the bundle less burdensome, it was suggested that the bundle should be adapted to incorporate or subsume current information‐gathering practices.

I think the idea is if we can incorporate things together, it will be a lot more appealing. So if this becomes a discharge summary, letter, then that will be very appealing, because they have to complete one anyway. So you'll get a lot more success if it's incorporated into this, rather than being a separate letter.

(Psychiatrist—Crisis Team)

#### 3.2.3 Integration Into Electronic Patient Record

To counteract these issues further, HCPs suggested the SAFER bundle needs to be integrated into the EPR system, rather than being a separate paper exercise to complete. Additionally, embedding the form into the EPR would benefit further with the ability to pull through information that has already been collected, such as demographic information, in other sections of the same system.

In my eyes, if there was a way that it could pull through information from one, so this could exist as a form, but you only have to input the information once and it could pull it through, then that to me would be better.

(Mental Health Nurse—Crisis Team)

However, HCPs did raise concerns with the accuracy of EPR systems to pull through the accurate updated information about a young person.

I guess some of that just depends on how neatly the information's put into the electronic patient record, if it's in the place that we've set up these bits to pull from. I think just the speed at which things happen within the crisis end of things, I think information's captured on the electronic patient record, but it's not always in the places where you've got things set up that auto‐pull.

(Mental Health Support Worker—Crisis Team)

### 3.3 Adaptations Required

SAFER‐YMH bundle was adapted using focus group data to create the SAFER‐YCL bundle. The focus group discussions highlighted that adaptations were required for the SAFER bundle to be successfully implemented in the Crisis–Liaison service context. As the original SAFER bundle was designed for the discharge process from inpatient units, HCPs identified specific areas requiring adaptation to fit with the approach of the Crisis and Liaison teams. These also included changes in terminology, for instance, changing the focus on admission to referral. A table of the adaptations made can be found in Supporting File S2.

#### 3.3.1 Crisis and Liaison Focus

HCPs identified additional elements that should be added to the bundle, including whether the young person is already involved with adult services if they are close to turning 18, identifying additional needs relating to religion, ethnicity or language and communication. On the other hand, due to the comprehensive nature of the bundle, HCPs felt several elements within the bundle should be optional, subject to individual HCPs' evaluation of whether certain elements (such as financial issues, neurodiversity) were pertinent to the young person.

#### 3.3.2 Timescales

Practically, HCPs also suggested a relaxation of the timescales associated with the bundle:

I think you'd be involving them in it regardless, I think it's just the timing. If they are at absolutely crisis point and you're going and trying to offer some containment, I think talking about discharge at that point would be really destabilising, so you would want just to think about the timing of when it was appropriate to do with the parent and the young person.

(Mental Health Nurse—Crisis Team)

#### 3.3.3 Optional Elements

The bundle was considered comprehensive in nature and would be time consuming to collect all the required information. As a result, HCPs suggested that certain elements were again made optional, such as boxes for medication, diagnosis, financial, accommodation or occupational needs.

#### 3.3.4 Simplicity

To alleviate other concerns around the length of the bundle, HCPs suggested further adaptations to allow for the implementation of the care bundle, such as the use of tick boxes (or drop‐down lists) rather than forms with large free‐text sections to fill out.

With a tick box I think it would be much easier, somebody is doing the liaison assessment, just tick box. Nobody's going to have the time, you have so much documentation to do already.

(Mental Health Nurse—Liaison Team)

#### 3.3.5 Additional Future Changes

The current format of the SAFER bundle is that of a paper form. Future adaptations require the three elements to be electronic forms incorporated into the EPR with the functionality to pull through existing information already held about a patient. They would then also be designed to supersede other documentation practices including the generation of safety plans and discharge letters.

## 4 Discussion

Stakeholder feedback was used to co‐adapt the SAFER‐YMH care bundle for CAMHS Crisis and Liaison, referred to as the SAFER‐YCL bundle. It identified the barriers and enablers to implementing this bundle in everyday practice.

From an NPT perspective, the bundle's implementation is more likely to succeed if other HCPs recognise its potential benefits for improving practice, patient experience and outcomes.

HCPs perceived the bundle as having the potential to replace existing data gathering processes, with its comprehensive social information capture adding value by consolidating key information from multiple domains. The inclusion of co‐produced elements, and its functionality as a handover or signposting document for other team members were acknowledged. This demonstrated participants' understanding of the bundle's purpose and value within the services (Coherence).

However, HCPs expressed concerns that the bundle might increase their workload, particularly regarding duplicated information gathering. Thus, the willingness of staff to be committed to using the bundle was questioned. They also questioned whether completing elements of the bundle could hinder the relationships they strive to develop and negatively impact the experiences of YP and their families during crises [19]. Changing practice and integrating a new intervention into the workload of a service (collective action) within the NHS is challenging, particularly when current services and staff already feel overstretched [20].

Some of these concerns may be alleviated by integrating the bundle into the EPR system, allowing for automatic pre‐population of elements if relevant data are available. Suggested amendments include making the bundle Crisis/Liaison focused, making elements optional and allowing flexibility in the timeframe for completion. These adjustments could mitigate concerns regarding duplication.

Whereas HCPs, YPs and PCs suggested flexibility in the completion timeframe, HPCs agreed that starting discharge planning early could reduce distress for patients receiving same‐day discharges after a multidisciplinary team (MDT) meeting. Thus, the initial element of the Patient and Workforce Discharge Pathway (PWDP) was to be conducted at admission, with subsequent weekly checks to keep patients informed [17].

The strengths of this study include the use of end‐user design incorporating diverse user points to optimise the bundle for clinical use. The methodology and analysis are grounded in real‐life practice, involving front‐line workers and stakeholders from across services, enhancing the feasibility and realism of the clinical impact and application of the findings. The multicentre initiative allows for testing the tools across different settings with varying practices and cultures.

However, the study has a few limitations. Participants were recruited from two NHS trusts, serving a population of approximately 2 million people. Given the differing models of practice among CAMHS Crisis and Liaison services across the country [21, 22], further adaptations may be required for the successful implementation of the bundle into other NHS trusts. A feasibility trial is needed in a larger number of trusts to fully understand the potential of the SAFER‐YCL bundle, as well as the implementation barriers and enablers identified during its use in practice. In addition, we found that focus groups held face‐to‐face tended to facilitate richer interaction due to the physical presence of participants, facilitators being able to pick up on non‐verbal cues. This was often somewhat lacking when held online, where interaction between participants was more fragmented and dominated by a few voices. Another limitation is that GPs, social services and community mental health teams—potential destinations for care after Crisis and Liaison—were not involved in these focus groups due to financial and time restraints. However, community care practitioners, such as General Practitioners (GPs), contributed to the initial development of SAFER care bundle, providing input on the information needed post‐discharge and what should or could be included in the social information capture section. Similarly, this study struggled to recruit YP and parents with experience of engaging with CAMHS Crisis and Liaison teams. As a result, their representation was limited, and the sample was skewed towards HCPs. It is likely that YP and parents who have recently experienced a mental health crisis may not yet feel ready to participate in research; it is also likely that those that have recently recovered from a crisis may not want to dwell on experiences during that time. Future research which uses a wider range of ways people can participate may be more successful in recruiting in this area.

The clinical implications of our findings highlight the potential of this tool to enhance the experiences of patients and clinicians during the transition of care between services, which is crucial given current pressures on these services and disparities that exist between services in relation to pathways out of these services [21, 22, 23]. If implemented successfully, this bundle could streamline YP's journeys through the Crisis–Liaison pathway, improving clinical effectiveness and cost efficiency, as well as the overall experiences of those who engage with these services [22]. This would also help maintain capacity for new cases by ensuring timely discharges and creating additional support for YP in crisis. As one of the key enablers identified by participants was the integration of the bundle into existing EPRs, future efforts should focus on how NHS digital platforms can host documents detailing a young person's CAMHS Crisis and Liaison journey and transition to other services.

## 5 Conclusions

The findings of this study, which utilised diverse stakeholder feedback, indicate that the SAFER‐YCL bundle has the potential to facilitate smoother transitions of care and optimise resource use. Feasibility testing is necessary to assess its effectiveness and inform future implementation. As CAMHS community services expand, evidence‐based service interventions like SAFER‐YCL are essential not only to support patient flow but also to improve their overall experiences.

## Author Contributions

**James Roe:** conceptualization, methodology, investigation, writing – original draft, writing – review and editing, formal analysis. **Neve Jones:** investigation, writing – review and editing, formal analysis. **Leonie Lewis:** investigation, writing – review and editing, formal analysis. **Natasha Tyler:** writing – review and editing, formal analysis, conceptualization. **Maria Panagioti:** formal analysis, writing – review and editing, conceptualization. **Sewanu Awhangansi:** writing – review and editing, formal analysis, investigation. **Nisha Balan:** writing – review and editing, formal analysis, investigation. **Nicola Wright:** formal analysis, writing – review and editing, conceptualization. **Richard Morriss:** funding acquisition, writing – review and editing, formal analysis. **Kapil Sayal:** funding acquisition, writing – review and editing, formal analysis. **Pallab Majumder:** writing – original draft, funding acquisition, conceptualization, investigation, methodology, writing – review and editing, formal analysis, supervision. **Josephine Holland:** funding acquisition, writing – original draft, investigation, conceptualization, methodology, writing – review and editing, formal analysis, supervision.

## Ethics Statement

Informed consent was appropriately obtained from all participants. East Midlands ‐ Nottingham 2 Research Ethics Committee provided ethical approval (REC reference23/EM/0274).

## Conflicts of Interest

The authors declare no conflicts of interest.

## Supporting information

Supporting Information.

Supporting Information.

## Data Availability

Ethics approvals do not allow for the publishing of full transcripts. Anonymous transcripts may be available on reasonable request.
