# Supplementary material for: Investigating the Applicability of the SAFER‐YCL Care Bundle for Transitions From CAMHS Crisis and Liaison Services: The Barriers and Enablers
Source: Health Expect. 2026 Feb 2;29(1):e70579. doi: 10.1111/hex.70579 (PMC12863418; doi:10.1111/hex.70579)
Supplement: Supplementary file 1 — Supporting Information. [file HEX-29-e70579-s001.pdf]

The University of Manchester

PATIENT CO-CREATED (WRITTEN) DISCHARGE PLAN (FOR CHILDREN AND PARENTS)

SECTION 1: Please complete 48 hours after admission or at the earliest appropriate time

Date completed: 02/17/2023

Preparing the patient for discharge

Things that might make me feel ready for discharge:

- My mental health has improved
- I feel confident, in myself, to be discharged
- I have had my physical health care needs reviewed
- When my admission is no longer suitable
- If I am out of my local area, I feel that my needs will be met
- We have discussed things that make me anxious about discharge

Things that will help me to feel ready for discharge:
[To be discussed and added here]

Preparing the parent/guardian for discharge

Things that might make my child feel ready for discharge:

- My child's mental health has improved
- We have the support to manage our child's risk
- My child's physical health care needs have been reviewed
- We have discussed things that make us anxious about discharge
- My financial responsibilities/difficulties that may affect my child's discharge have been discussed
- If we are out of my local area, we feel that our child's needs will be met

Things that will help me to feel ready for my child's discharge: [e.g., Someone to talk to when problems arise; right package of support; therapeutic programme on practical skills; digital meetings with other parents]
[To be discussed and added here]

PLEASE TURN OVER >

SECTION 2: Please complete within 24 hours before discharge

Date completed: 02/17/2023

Things that matter to me (and my parent/guardian) after discharge

What matters most to me (us) is:

My (our) main concern is:

Information I (we) need but don't have:

What will help me prevent another admission:

Brief summary of diagnosis and formulation:

These activities will be good for my health and wellbeing:

SECTION 2 continued

My appointments

My next health care appointment:

Date: 02/17/2023	Time:	Place:
With:		Phone number:
Reason for appointment:		

My next educational appointment:

Date: 02/17/2023	Time:	Place:
With:		Phone number:
Reason for appointment:		

My next social care appointment:

Date: 02/17/2023	Time:	Place:
With:		Phone number:
Reason for appointment:		

Questions to ask at next appointment: (e.g., medication, treatment continuation at home, do they feel things are going in the right direction, is there anything worrying you?)

At my next appointment I could ask:

Other feelings I might have and what to ask:

Concerns about my next appointment:

Then my future appointments are:

If I have a problem after discharge, I can call:

Name:	Phone number:	Out of hours:

Additional contact information of people that can help me after discharge:

Name:	Phone number:	Out of hours:
Name:	Phone number:	Out of hours:
Name:	Phone number:	Out of hours:

PLEASE TURN OVER >

SECTION 2 continued

Medicines

I/my child should take these medications for their mental health:

**Medication 1 name:**	Generic name:
How much to take:	
How many times a day to take it:	
How to take it:	
How it can help me:	

**Medication 2 name:**	Generic name:
How much to take:	
How many times a day to take it:	
How to take it:	
How it can help me:	

**Medication 3 name:**	Generic name:
How much to take:	
How many times a day to take it:	
How to take it:	
How it can help me:	

**Medication 4 name:**	Generic name:
How much to take:	
How many times a day to take it:	
How to take it:	
How it can help me:	

I/my child should take these medicines for their physical health:

Generic name:
How much to take:
How many times a day to take it:
How to take it:
How it can help me:
Other medicines that my child can or can't take:
Medication allergies:

SECTION 2 continued

Additional notes

MANCHESTER 1824
The University of Manchester

SOCIAL INFORMATION CAPTURE AT ADMISSION

Date completed:

Complete within 24 hours of admission or the earliest appropriate time

Personal details:

Name:

DOB: | Sex: | Gender, if different from sex:

Contact details:

Phone: | Email:

Estimated discharge date:

Date:

Safeguarding status:

(e.g., what has been put into place to protect the patient)

Care giving responsibilities:

Parent/guardian 1:

Name:

Phone: Consent ☐

Email: Consent ☐

Relationship to patient:

Parent/guardian 2:

Name:

Phone: Consent ☐

Email: Consent ☐

Relationship to patient:

Care coordinator:

Name:

Phone:

Email:

Social worker:

Name:

Phone:

Email:

School contact:

Name:

Phone:

Email:

Physical healthcare needs:

(e.g., chronic health conditions, addictions, history/prescriptions)

Accommodation status:

(e.g., do they have permanent accommodation, is it fit for habitation, gender appropriate, safe/secure)

PLEASE TURN OVER >

GP name and address:

GP Name:

GP Address:

Postcode:

Family financial responsibilities:

(e.g., unmanageable debt, unofficial debt, receipt/registration for benefits)

Benefits status:

(e.g., have they been entitled to help from the government?)

Unmet needs in the community:

(e.g., isolation, participation in meaningful activities, employment/studying)

Education status:

(e.g., school, college, work or not doing anything)

Any neurodiversity:

(autism, ADHD, dyslexia)

If yes, do they have Care, Education, and Treatment Reviews (CETR) before they were admitted?

Involvement with other professional and social care services:

Placement needs to be considered after discharge:

Support that family may need:

MANCHESTER 1824

The University of Manchester

SAFER-YMH CHECKLISTS

Section 1: Please complete within 48 hours after the Social Information Capture form

Parent/guardian check

Name of contact:	Action:
Date:	
Name of ward staff:	

Safeguarding check (e.g., do they have social support and care already in place?)

Name of contact:	Action:
Date:	
Name of ward staff:	

Accommodation check (e.g., do they have housing in place?)

Name of contact:	Action:
Date:	
Name of ward staff:	

Financial check (e.g., do they have any debts or are they receiving any benefits?)

Name of contact:	Action:
Date:	
Name of ward staff:	

Social care/social work check (e.g., have they been allocated to social worker/carer?)

Name of contact:	Action:
Date:	
Name of ward staff:	

Placement needs check

Name of contact:	Action:
Date:	
Name of ward staff:	

Develop criteria for discharge (e.g., personal goals to achieve)

Name of contact:	Action:
Date:	
Name of ward staff:	

Introduce/check the patient written discharge plan

Name of contact:	Action:
Date:	
Name of ward staff:	

PLEASE TURN OVER >

Section 2: Please complete/check weekly after completing section one until discharge

Revisit criteria for discharge and estimated discharge date

Name of contact:	Action:
Date:	
Name of ward staff:	

Revisit patient written discharge plan (e.g., are any amendments needed)

Name of contact:	Action:
Date:	
Name of ward staff:	

Accommodation check (e.g., safety of house, carer/family, risks in community)

Name of contact:	Action:
Date:	
Name of ward staff:	

Placement needs check

Name of contact:	Action:
Date:	
Name of ward staff:	

Family support needs check

Name of contact:	Action:
Date:	
Name of ward staff:	

Other services (not mentioned above) contacted

Name of contact:	Action:
Date:	
Name of ward staff:	

Please record any additional actions completed/ to be completed regarding the above in this box

Action:

Section 3: 48-72 hours before discharge

Confirm with consultant that medications are prepared

Name of contact:	Action:
Date:	
Name of ward staff:	

Confirm appropriate housing in place

Name of contact:	Action:
Date:	
Name of ward staff:	

Confirm that community services have acknowledged the discharge

Name of contact:	Action:
Date:	
Name of ward staff:	

Section 4: In 24 hours before discharge

Complete patient written discharge plan and give a copy to the patient

Name of contact:	Action:
Date:	
Name of ward staff:	
