# Supplementary material for: Investigating the Applicability of the SAFER‐YCL Care Bundle for Transitions From CAMHS Crisis and Liaison Services: The Barriers and Enablers
Source: Health Expect. 2026 Feb 2;29(1):e70579. doi: 10.1111/hex.70579 (PMC12863418; doi:10.1111/hex.70579)
Supplement: Supplementary file 2 — Supporting Information. [file HEX-29-e70579-s002.docx]

Additional file 2: Summary of required adaptations

| SAFER-YMH Bundle Element | Adaptation(s) Required |
| --- | --- |
| Social Information Capture | Addition of involvement with adult services  Addition of patient preferred pronouns, ethnicity  Parent/guardian 2 as optional  Addition of parent/guardian address  Addition of parental responsibility  Combining financial responsibilities and benefits status  Addition of Recent or current family health/mental health issues as optional element  Addition of Ethnicity/religious/spiritual/language needs as optional element  Addition of intellectual difficulties to neurodiversity element  Addition of Communication needs  Change following elements as optional:   - Discharge date - Financial issues - Unmet needs in the community - Any neurodiversity/intellectual difficulties - Involvement with other services - Placement needs |
| Patient Co-created (Written) Discharge Plan | Name change to Patient Co-created (Written) Discharge and Safety Plan  Removal of “When my admission is no longer suitable”  Additional check to confirm young person/child is happy to be able to view parent/carer comments  Brief summary of formulation – diagnosis removed  Social activities added to “these activities will be good for my health and wellbeing”  Addition of “How can I tell if I am feeling unwell again (include recognition of triggers/signs of a relapse)”  Addition of “Steps I need to take to keep myself safe if I start to feel unwell”  Addition of “What parent/carer(s) need to do to keep patient safe (include changes at home)”  Addition of “Summary of Crisis/Liaison journey (clinician copy) and option to make available to Patient, GP, Parent/Carer”  Appointments and questions to ask at appointments now optional  “Additional contact information of people that can help me after discharge” changed to “Additional contact information of people that I feel safe with (include services, teachers etc.)”  Medication section optional |
| Checklists | Instructions changed to “Please begin as soon as possible after the Social Information Capture form or at the earliest appropriate time, and complete during discharge planning phase”  Removal of “name of contact”  “Name of ward staff” changed to “Agency/team flagged to”  Addition of “any issues flagged from Social Information Capture” to Section 1 elements (apart from Safeguarding element)  Addition of “Appropriately signposted” to Section 1 elements (apart from Safeguarding element)  Removal of “ward staff” from Safeguarding element  All elements within Section 1 and 2 (apart from Safeguarding) made optional  Accommodation check information changed to “check for changes in living arrangements”  Addition of “Autism support” to “other services” element in Section 2  Section 3 made optional  Section 4 incorporated not Section 3 |
| All Elements | Reference to “admission” changed to “referral” or “crisis team support” where relevant |
